# Evolution of Phase Transformations in the Mg-Ni-Ce System After Mechanical Synthesis and Spark Plasma Sintering

**DOI:** 10.3390/ma18092131

**Published:** 2025-05-06

**Authors:** Nuriya Meiramkanovna Mukhamedova, Arman Zhanarbekovich Miniyazov, Gainiya Kaiyrdykyzy Zhanbolatova, Zhanna Nurbolatovna Ospanova, Aisara Askhatkyzy Sabyrtayeva, Karina Serikkyzy Shaikieva

**Affiliations:** Institute of Atomic Energy Branch of the National Nuclear Center of the Republic of Kazakhstan, 10 Beybit Atom Str., Kurchatov 071100, Kazakhstan; bakayeva@nnc.kz (N.M.M.); miniyazov@nnc.kz (A.Z.M.); kaiyrdy@nnc.kz (G.K.Z.); sabyrtayeva@nnc.kz (A.A.S.); shaikieva@nnc.kz (K.S.S.)

**Keywords:** mechanical synthesis, phase transformations, Mg-Ni-Ce alloy, X-ray phase analysis, Scherrer method

## Abstract

The present study focuses on investigating the evolution of phase transformations in the Mg-Ni-Ce system under the influence of mechanical synthesis (MS) and spark plasma sintering (SPS). Magnesium powder mixtures with different nickel and cerium contents (Mg-3%Ni-2%Ce, Mg-7%Ni-3%Ce, and Mg-10%Ni-5%Ce) were mechanically activated along with various grinding parameters. The X-ray phase analysis (XRD) has shown the successive stages of the phase formation in the MS process: from the initial components to the formation of intermetallic compounds of Mg_2_Ni, Mg_12_Ni_6_, and CeMg_3_. An increase in the intensity of mechanical treatment facilitated the accelerated destruction of the crystal lattice, the generation of defects, and the formation of new phases, as evidenced by the broadening and reduction in the intensity of Mg diffraction peaks. The subsequent SPS stage promoted the completion of phase transformations, structural stabilization, and the formation of a dense, multicomponent microstructure with a uniform distribution of intermetallic compounds. The observed average crystallite sizes ranged from 20 to 70 nm, depending on the processing conditions. The research results demonstrate the possibility of targeted control over the phase composition, opening new opportunities for the development of highly efficient hydrogen-absorbing alloys.

## 1. Introduction

Currently, metal hydrides are being widely studied for hydrogen storage, among which magnesium-based compounds (Mg) appear promising due to their high hydrogen capacity (a theoretical value of up to 7.6 wt.%), availability, and environmental safety [1,2,3]. Despite the fact that magnesium hydride (MgH_2_) is one of the few metal hydrides capable of reversible hydrogenation and dehydrogenation, its practical application is limited by the low kinetics of these processes [4,5,6,7,8]. The kinetic mechanism of the interaction between Mg-based intermetallic compounds and hydrogen, which includes several key stages, is described in the studies of [6,7]. At the initial stage, hydrogen molecules are adsorbed on the surface of Mg, followed by their dissociation. Then, atomic hydrogen penetrates through the solid surface and diffuses into the material. Subsequently, a solid solution of Mg-H (α-phase) is formed, which, upon reaching a certain saturation level, transforms into magnesium hydride (β-phase) [8].

It should also be noted that the low resistance to atmospheric contaminants, particularly the oxidation of the magnesium surface, hinders not only the dissociation process but also the subsequent diffusion of atomic hydrogen, as well as leads to the rapid degradation of the material’s sorption characteristics [9,10]. Methods for improving the kinetics of hydrogen sorption in Mg-based materials include alloying with transition metals [11,12], Mg nanostructuring by the MS in grinding mills [13], and intensive plastic deformation [14,15].

It was noted in [15] that Ni is an effective catalytic element in Mg-based alloys. Charging the Mg–Ni alloys with hydrogen leads to the formation of several phases containing hydrogen: a hydrogen solid solution in Mg (HCP) and Ni (FCC), as well as in MgH_2_ hydrides (tetragonal at 1 bar) and Mg_2_NiH_4_. It has been found that their presence in the Mg_2_NiH_4_ compound has better kinetics of hydrogen absorption and desorption than the pure Mg_2_Ni compound. It was revealed in [16,17] that the inclusion of rare-earth elements (REs) in the Mg-Ni (Mg-Ni-RE) system makes it possible to improve the complex properties of hydrogen storage due to their highly dispersed structure and the synergistic effect between Ni and RE. REs are also well-known oxygen absorbers, which results in the improved resistance to the Mg oxidation and hence improved hydrogen absorption [18,19]. Cerium (Ce) is considered to be one of these REs, which not only reduces the formation of surface Mg oxides but also forms composites with faster hydrogen sorption kinetics [20,21]. The studies by Leiva [22] and Yartys [23] have shown that the addition of Ce forms Mg_3_Ce alloys, which can absorb hydrogen, forming MgH_2_ hydride even at room temperature due to the synergistic effect of the hydrides formed in situ, improving the material kinetic properties.

The MS method has been widely utilized for obtaining Mg (Mg-Ni-RE)-based intermetallic compounds. This approach enables the production of nanoscale particles with a predetermined chemical composition and microstructure through prolonged mixing of the initial powders with alloying elements in planetary ball milling systems [24,25]. This approach makes it possible to reduce the size of crystals, which significantly increases the area of the material’s active surface and the number of defects in the crystal lattice, which play an important role in the hydride formation processes [26]. It was shown in [27,28] that the use of mechanical synthesis provides nanostructured powders with improved characteristics and significantly increases the reactivity of magnesium hydrides, including an increase in the hydrogen absorption kinetics. Nevertheless, it is important to note that parameters such as the ball-to-powder ratio (BPR), the duration, and the rotation speed of mechanical synthesis significantly influence the final properties of the material. Yan [29] and the coauthors note that when Ce_5_Mg_85_Ni_10_ is obtained, the BPR of 20:1 helps to reduce the size of crystallites and form lattice defects necessary to improve the hydrogen absorption kinetics. At the same time, ref. [30] shows that higher BPR values (30:1) result in the formation of excessive amounts of magnesium oxides, which reduce the effectiveness of the materials.

The activation of the sintering process of powder compositions (PCs) with pulsed current while applying an external pressure, also known as SPS technology, which has been actively studied for the recent decades, opens up an opportunity for solving this problem [31,32,33,34].

The combination of MS and SPS methods opens up opportunities for the consolidation of intermetallic alloys. However, the study of the effect of the sequential application of the two methods on the structure and composition of the powder material is at an early stage. Recent literature reviews have noted that many of the processes occurring in MS and SPS are not yet clear enough [35].

In this regard, the goal of this work was to establish the patterns affecting the MS and further SPS parameters on the structure and formation of intermetallic phases in the Mg-Ni-Ce system for creating the functional materials for hydrogen storage and transportation.

## 2. Materials and Methods

The initial materials used to study phase formation and structural changes in the Mg-Ni-Ce system, resulting from a sequential combination of MS and isostatic pressing (IP) for metal hydride production, were high-purity metallic powders of Mg, Ni, and Ce. These included magnesium powder (GOST 6001-79, grade MPF-3, purity 99%, particle size 120–180 µm), metallic cerium (purity 99.99%, particle size 70 µm), and carbonyl nickel powder (grade PNK-UT3, purity 99.99%, particle size 20 µm), supplied by Suoyi, Handan, Hebei Province, China.

PMs were prepared using the planetary ball mill Retsch PM100 (Haan, Germany) via the MS method. Depending on the component ratio, all samples were divided into three batches, with 12 samples in each. The conditions for PM preparation are presented in Table 1. The selected ratios were based on a theoretical analysis of previous studies on the influence of alloying elements on the formation of intermetallic compounds.

The amount of the powder was calculated taking into account the mass of the grinding balls used. In all experiments, the same balls with a total mass of 385 g and a volume of 80–83 mL were used, which approximately corresponds to one-third of the milling pot volume. During the experiments, a milling chamber with a capacity of 250 mL was used, containing 5 mm stainless-steel milling balls made of grade 1.4034/AISI 420. To prevent oxidation, powder preparation, weighing, and loading of the powder–ball mixture into the milling chamber were carried out in a glove vacuum box (GVB-3C) under an argon atmosphere. The rate of the MS and its duration changed, which amounted to 350 and 450 rpm, with 5 and 10 h, respectively, for each group of BPR samples. The rotation direction was changed every hour during the MS for even redistribution of the particles and to reduce the likelihood of their gluing.

The sintering of the powders was carried out using the SPS CY-SPS-T20 (High-tech Zone, Zhengzhou, China). The process took place in several stages: After the MS, the PCs were placed in a graphite mold with a working diameter of 25 mm in a vacuum glove box VGB-3C, in an online argon medium with an excess pressure of 5 kPa, and pre-pressed (1–2 MPa); then, the mold was placed in a sintering chamber, vacuumized (up to 1 Pa), and then sintered. To avoid baking onto the walls of the mold and to the punches, the PC was insulated from them with the graphite paper. The volume reduction was at a pressure of 1–2 MPa, an exposure time of 5 min, and a heating rate of 100 °C/min, and was under natural cooling conditions. The sintering temperature was 560 ± 10 °C. The temperature of the SPS process was controlled by an optical pyrometer focused on an opening located in the middle of the plane of the outer wall of the mold with a depth of 5.5 mm.

The analysis of the phase and elemental composition was conducted both after the preparation of the PC using the MS method and after obtaining the sintered samples through SPS. X-ray diffractograms of the samples were obtained by an EMPYREAN diffractometer from Panalytical with Cu-Kα radiation (Almelo, The Netherlands). The voltage and current were set to 45 kV and 40 mA, respectively. The exposure time (time per step) during the shooting was 30.6 s, the scanning step size for the diffractograms was 0.013°2θ, and the studied angular range was 5–153°2θ. A fixed divergence slit with an angular divergence of 1° and an incident beam mask marked 15 were used. The diffractograms were processed in the angular range of 20–90°2θ in the HighScore program, which was designed for phase analysis and identification, using PDF-4 AXIOM databases. The crystallite size was determined using mathematical calculations based on the Scherrer equation:

(1) $$D=\frac{K\lambda }{\beta \cos\theta \;}\;,$$

where *D* is the crystallite size, *K* is the shape factor, *λ* is the wavelength of the X-ray radiation, *β* is the full width at half maximum (FWHM) of the peak, and *θ* is the diffraction angle. The peak widths were determined by fitting a Gaussian function in the HighScore software. Taking into account the same conditions of the diffraction experiment, the visual differences in the diffractogram indicate the differences in phase composition and structure. When applying the diffractometric data to the diffractograms of the cards, the experimental peak intensities did not completely match the barcode of the card used, despite the complete correspondence of the angular positions. In this regard, to identify the phase composition, preference was given to the cards that are most suitable in the angular positions and have the highest compliance rating.

## 3. Results and Discussion

**Figure 1:**
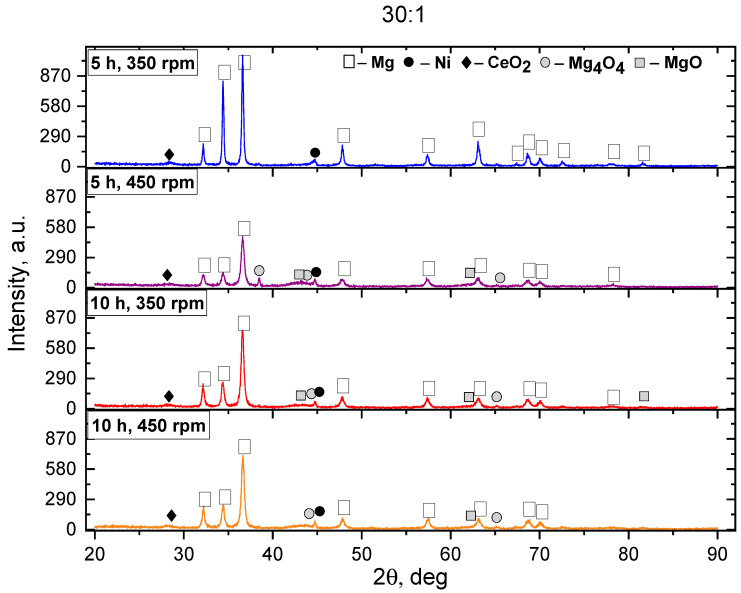
Diffractograms of the studied PC of batch I after the MS: the ratio of the mass of the grinding balls to the mass of the PC is 30:1.

The mechanical synthesis made it possible to achieve significant changes in the phase composition of the Mg-Ni-Ce system samples. According to the XRD results, it was found that after the MS, the basis of the phase composition of all samples of batch I (Mg-3%Ni-2%Ce) was Mg, which was characterized by a hexagonal crystal lattice, a P-63/mmc spatial group, and lattice parameters of a = b = 3.212 Å and c = 5.215 Å. When analyzing the obtained diffractograms, it was found that after the MS, the Mg active oxidation occurs at BPRs of 10:1 and 20:1, which was confirmed by the peaks belonging to the Mg_4_O_4_ and MgO oxide phases. This is due to the mechanochemical interaction with oxygen when fewer milling media are used. Conversely, increasing the ball-to-powder mass ratio to 30:1 also promotes the formation of CeO_2_. Consequently, for further analysis, the superposition of the diffractograms from the samples of the first batch after the MS with a BPR of 30:1 is shown in Figure 1.

The diffractograms were obtained from the samples after the MS showed a decrease and broadening of the peaks belonging to the Mg phases and their shift to a small-angle region, which indicates a decline in the crystallinity degree, an increase in the number of defects in the crystalline structure, and the formation of internal stresses. In the case of the MS, this facilitates the formation of the new phases, which is confirmed by an increase in the intensity of peaks belonging to the oxide phases [21,36,37,38,39]. The change in the rotation rate during the MS has a special effect on the decrease in intensity and broadening. This is due to the increased mechanical energy transferred to the powder particles and friction during the synthesis process. The presence of Ce oxides, according to [40,41,42,43,44], should reduce the content of Mg oxides on the surface and indicate an improvement in the oxidation resistance due to microstructural changes during contact with the air. However, as can be seen from the diffractograms obtained from the samples of batch I, the peaks were observed corresponding to the phases of the Mg oxides, the intensity of which is higher than that of the Ce oxide.

**Figure 2:**
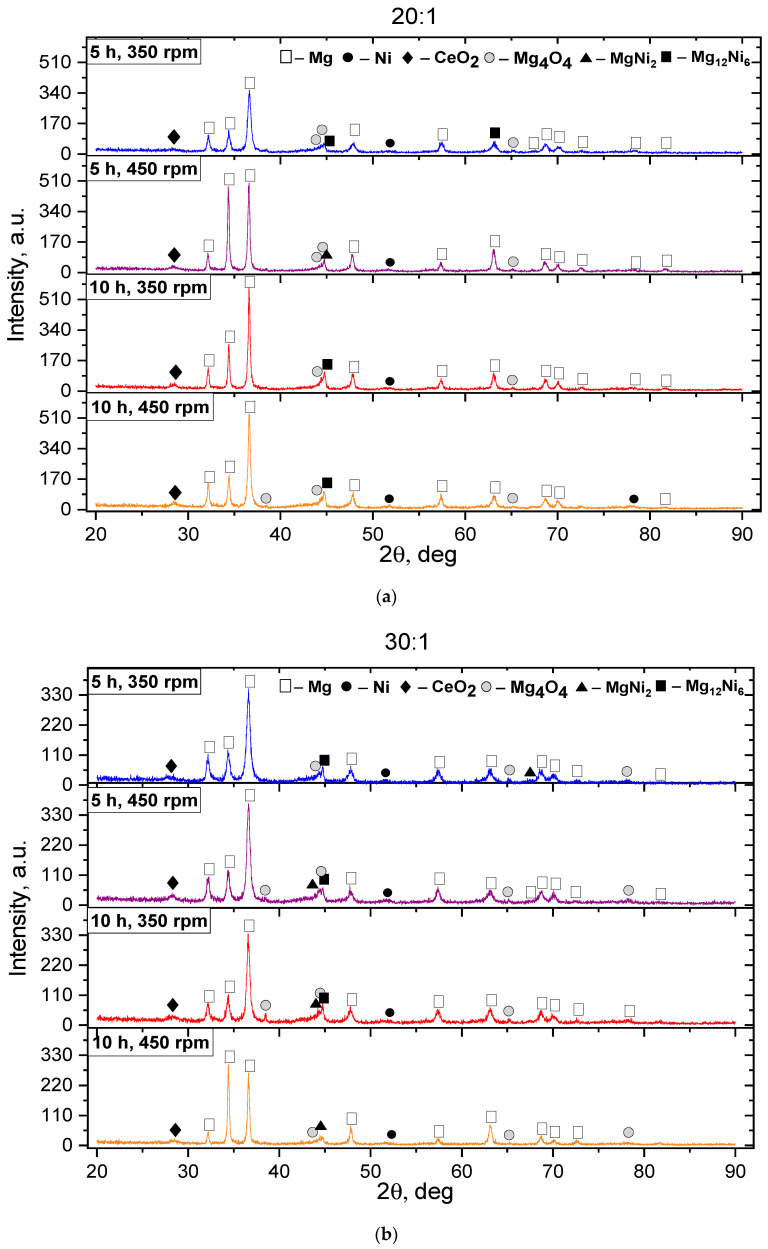
Diffractograms of the studied PC of batch II after the MS. The ratio of grinding balls to PC: (**a**) 20:1 and (**b**) 30:1.

According to the XRD results, it has been found that the BPR of 10:1 contributes to a slight decrease in the size of crystallites and the formation of only oxide phases. Consequently, a further analysis of the effect of MS conditions on the formation of intermetallic phases in the Mg-Ni-Ce system was conducted using diffractograms obtained from samples with a ball-to-powder mass ratio of 20:1 and 30:1. Figure 2 presents the X-ray diffractograms of batch II PS after MS at different milling durations and intensities.

**Figure 3:**
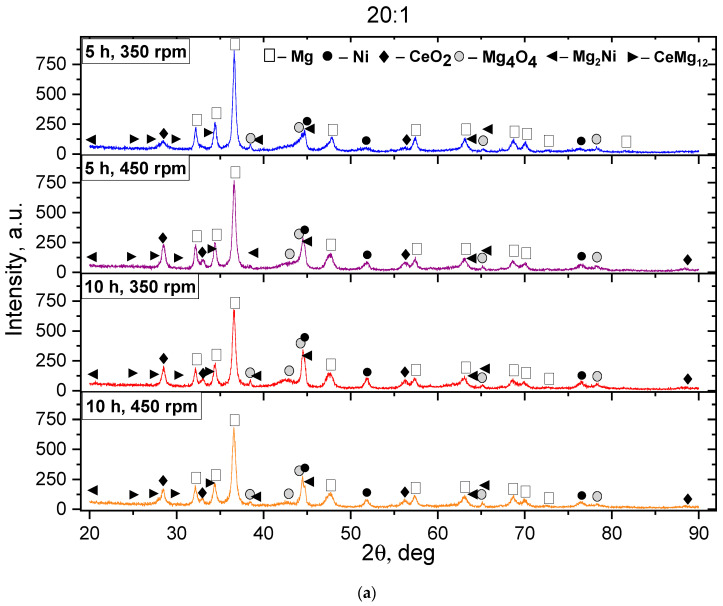

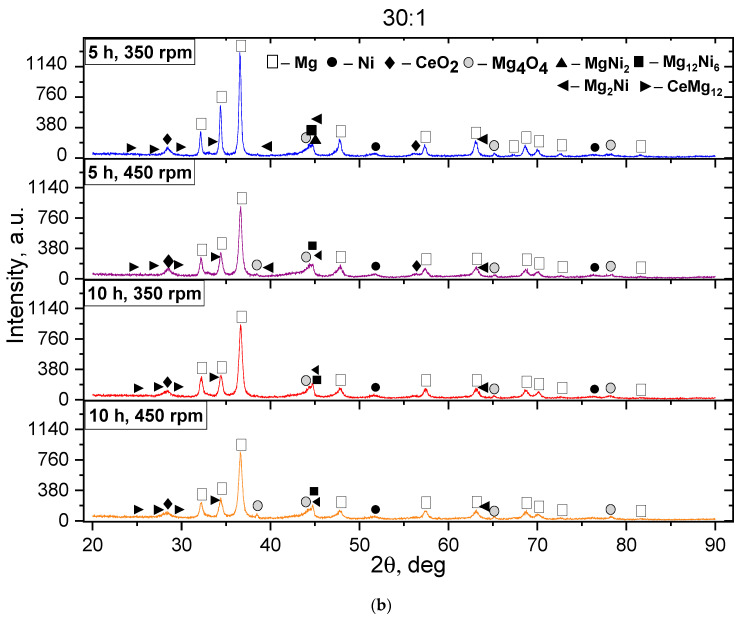
Diffractograms of the studied PC of batch III after the MS. The ratio of the mass of the grinding balls to the mass of PC: (**a**) 20:1 and (**b**) 30:1.

There is a general decrease in the peak intensities relative to batch I. The Mg, characterized by a hexagonal crystal lattice (P-63/mmc), remains the basis of the phase composition of all samples after the MS. A higher ratio of the mass of the milling balls to the mass of the PS leads to a decrease in the intensity and broadening of the Mg peaks, inducing their shift to the region of large angles, which indicates the formation of new phases based on the Mg. This is evidenced by low-intensity peaks corresponding to the intermetallic phases of MgNi_2_, Mg_12_Ni_6_, and MgNi_2_, along with the oxide phases of CeO_2_ and Mg_4_O_4_. Nevertheless, the peaks shifted parameters towards smaller angles. The displacement of the Mg peaks is caused by a decrease in the FCC lattice parameter when the Mg atoms are replaced by the Ni atoms, which is confirmed by the observed decrease in the intensity of the Ni peaks with an increase in the MS duration. It should also be noted that after mechanical alloying (MA) for 10 h at an acceleration of 450 rpm, a higher BPR reduces the formation of Mg oxides. The diffraction peaks become broader due to an increase in internal deformation and a decrease in grain size. This suggests that the samples belong to a nanocrystalline structure [45], which is confirmed by the crystallite size data presented below in Table 2. The imposition of the diffractograms of the PC batch III after the MS is shown in Figure 3.

The basis of the phase composition of all batch III samples, as in the first two, is the Mg, characterized by a hexagonal crystal lattice and a P-63/mmc spatial group. Low-intensity peaks belonging to the oxide phases of Mg_4_O_4_ and MgO are observed. However, the presence of more Ce in the initial charge has led to an improvement in the resistance to the Mg oxidation and a decrease in the content of Mg oxides, as evidenced by the appearance of high-intensity diffraction peaks relative to batches I and II belonging to CeO_2_. A comparison with the samples of batches I and II showed that the diffractograms of batch III contain high-intensity reflections peculiar to Ni, which is associated with the high nickel content. The Ni diffraction peaks, with an increasing MS rate, have shown broadening and a decrease in intensity, indicating the formation of new phases. The presence of large amounts of Cu and Ni leads to the formation of new intermetallic phases of MgNi_2_, Mg_12_Ni_6_, Mg_2_Ni, and CeMg_12_, which is evidenced by the appearance of the diffraction peaks. It is worth noting that the mass ratio of the grinding balls to the mass of the powder mixture, equal to 30:1, helps to reduce the Ni content and form new phases of the Mg–Ni system. Charging the Mg–Ni alloys with hydrogen leads to the formation of several phases containing hydrogen [36,45], including MgH_2_ and Mg_2_NiH_4_. It was found that the Mg_2_NiH_4_ compound has better kinetics of hydrogen absorption and desorption than the pure Mg_2_Ni compound. According to [26], Mg_2_NiH_4_ not only has better kinetics of hydrogen absorption and desorption but also a lower desorption temperature (223 °C) at an equilibrium pressure of 0.1 MPa.

As it was noted earlier, an increase in the duration and rate of the MS facilitates a decrease in the size of crystallites and the formation of lattice defects necessary to improve the hydrogen absorption kinetics. To estimate the degree of milling of the phases we are interested in after the MS, the sizes of all crystallites were calculated using the Scherrer method. Table 2 shows the calculation results.

The calculations show that after mechanical treatment, there is a significant reduction in crystallite size, which is also confirmed by the broadening of the diffraction peaks. The smaller crystallites are observed in the newly formed phases of CeMg_12_ (27.58 nm) and Mg (29.85 nm), which indicates a high defect and active mechanical destruction of the crystal lattice. The smallest crystallites are observed in the CeMg_12_ (27.58 nm) and Mg (29.85 nm) newly formed phases, which indicates a high defect and active mechanical destruction of the crystal lattice. On the contrary, the newly formed Mg_2_Ni (38.84 nm) and Mg_12_Ni_6_ (45.73 nm) phases after the MS are characterized by larger crystallites, which reflects the peculiarities of their formation during the mechanical treatment. A comparative analysis of the sizes of magnesium crystallites under various studies reveals patterns related to mechanical synthesis and its parameters. Thus, in the work of Hwang et al. [46], the size of the magnesium crystallite was 42 nm, which was slightly larger than in our study. In the study of Sutapa [47], it reached 10 nm, which was noticeably less than that of all batches in the present work after the mechanical treatment. In turn, Ratner et al. [34] recorded a wide range of sizes of magnesium crystallites from 26 to 71 nm.

The analysis of the data from the three batches shows that the size of the magnesium crystallites varies depending on the mechanical treatment conditions. In batch III, where the smallest CeMg_12_ (27.58 nm) and Mg (29.85 nm) crystallites were obtained, the high defects and active mechanical destruction of the crystal lattice were observed. At the same time, the first two batches also showed a decrease in crystallite sizes, but to a lesser extent, which may be due to differences in the MS parameters.

A comparison with literature data reveals a significant correlation between crystallite size and reactivity. Smaller crystallites exhibit a larger active surface area, which promotes the formation of hydride phases. However, if the crystallite size becomes too small (below 10–20 nm) [34], surface oxidation intensifies, necessitating the optimization of mechanical treatment parameters and subsequent sintering to regulate the composition and structure of the resulting phases. Thereby, it is important to take into consideration the surface oxidation effect, especially with the small particle sizes, and correct the MS parameters to minimize it, since after the SPS, the intermetallic phases dominate in the samples, which is confirmed by the appearance of the pronounced peaks of Mg_2_Ni, Mg_12_Ni_6_, and CeMg_3_.

**Figure 4:**
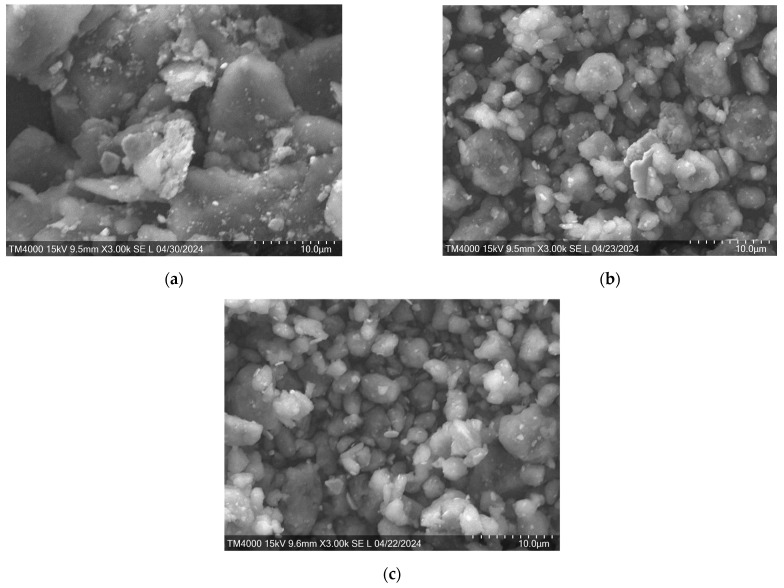
SEM morphologies of the milled PC of batch III after the MS. The ratio of the mass of the grinding balls to the mass of PC: (**a**) 10:1, (**b**) 20:1, and (**c**) 30:1.

It should also be taken into account that the state of the particle surface also has a great influence on the decomposition of hydrogen molecules [48]. Figure 4 shows the morphology of the PS after MS.

As can be seen from Figure 4, during ball milling, an increase in the ball-to-powder mass ratio leads to a significant decrease in particle size. The PS after MS at BPR 10:1 has a dendritic (flaky) shape. The PS particles after MS at a BPR of 20:1 have both a dendritic and an oval shape. It is worth noting that the PS particles after MS at BPRs of 20:1 and 30:1 have a smooth surface. The formation of the powder structure during MS helps to improve the interaction between particles in the Mg-Ni-Ce system by increasing the specific area of interaction of the elements, which plays a key role in the subsequent SPS [49].

**Figure 5:**
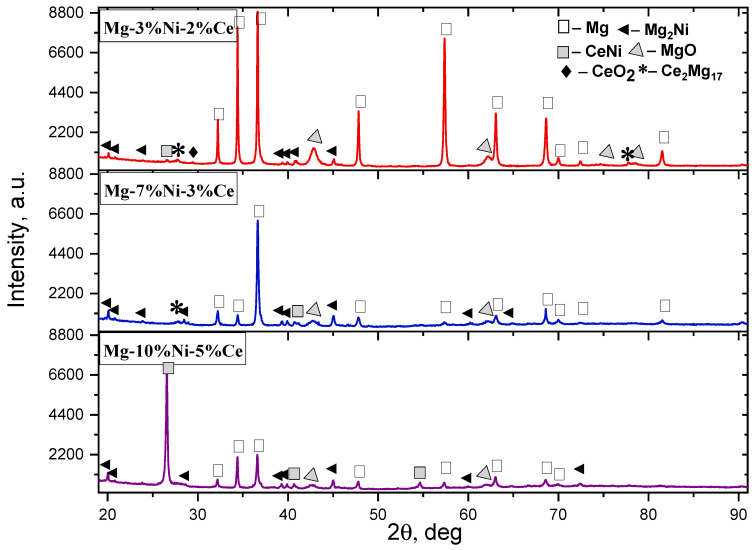
Imposition of diffractograms of samples of Mg-3%Ni-2%Ce, Mg-7%Ni-3%Ce, and Mg-10%Ni-5%Ce after SPS.

After the SPS, the presence of the CeNi compound in the Mg-10%Ni-5%Ce sample was recorded on the diffractograms above, whereas its presence was not confirmed after the mechanical treatment. This is probably due to the increased content of Ce and Ni in the alloy composition. The high degree of crystallinity of these phases has been confirmed by the presence of narrow and well-resolved peaks in the diffractograms shown in Figure 5, which indicates a high order of the crystal lattice. When analyzing the diffractograms of the three samples in Figure 5 taking into account the identical XRD conditions, the structural changes related to the percentages of the alloying elements in the samples are noticeable. At a temperature of 500–560 °C, the components were evenly distributed, which prevented the formation of large oxide phases.

Based on the X–ray phase analysis (SPS) of the sintered samples, it was found that the Mg-3%Ni-2%Ce sample was characterized by high and narrow peaks of Mg phases.

In the Mg-7%Ni-3%Ce sample, a decrease was observed in the intensity of the fractional peaks of the Mg and Ni phases, as well as a pronounced shift in the reflection, which indicates the possible formation of the intermetallic compounds.

The diffraction patterns of the sintered compacts of the Mg-10%Ni-5%Ce sample demonstrated the appearance of several additional reflections characteristic of the CeNi intermetallic compound. The data analysis indicated a regular increase in the number of metal hydride phases with an increase in the content of the alloying elements, which confirms the active participation of Ni and Ce in the structural changes in the Mg matrix. This is probably due to the completion of the CeNi formation during the SPS, which was accompanied by an increase in the size of crystallites during the recrystallization because of an increase in temperature during the SPS. At a low Ni content (3%), the Mg_2_Ni phase is mainly formed, which is confirmed by the appearance of reflections in the area of 30°, 41°, 45°, and 60°. When the Ni content increases to 7–10%, the Mg_12_Ni_6_ and MgNi_2_ phases are formed. This is due to the high solubility of Ni even at the MS stage, which is conditioned by the increased thermodynamic stability of these compounds [29]. Due to this, they remain even after the SPS stage. Such changes, along with the predicted formation of the hydride phases, contribute to improving the kinetics of the sorption processes. The effect of Ni and Ce alloying on the phase composition and crystallite size of the Mg-based alloys is shown in Table 3. Ce facilitates the formation of the dispersed SeMd_3_ and SeMd_12_ intermetallides, which act as barriers to the agglomeration of the Mg grains, preventing undesirable agglomeration of the particles.

A decrease in the size of crystallites and the formation of more complex intermerallide phases is observed with an increase in the concentration of the alloying components, which has a positive effect on the material’s mechanical and hydride properties, confirming the obtained experimental results [50]. The most stable structures are observed at a content of Ce-3% and Ni-7%, while achieving an optimal balance between the size of the crystallites, phase composition, and mechanical properties.

The largest increase in the crystallite sizes is observed in Mg_2_Ni (from 38.8 nm after the MS to 55.64 nm after the SPS) and Mg_12_Ni_6_ (from 45.73 nm after the MS to 69.64 nm after the SPS), which confirms the stabilization during the sintering. Mg also shows an increase in the crystallites, but to a lesser extent than the intermetallides (from 29.85 nm after the MS and up to 46.45 nm after the SPS). CeMg_3_ (from 27.58 after the MS to 37.42 nm after the SPS), Ce_2_Mg_17_ (from 33.62 after the MS to 42.90 nm after the SPS), and Ce_2_Mg_17_ (from 33.62 nm after the MS to 42.90 nm after the SPS) increased, but remained more dispersed, which may be due to their thermodynamic stability and high melting point. The increase in the size of the Mg_2_Ni and Mg_12_Ni_6_ phases indicates their recrystallization and the formation of larger grains, which contributes to the increased structural stability during the cyclic hydrogenation and dehydrogenation processes, as noted in [25].

## 4. Conclusions

As a result of the study of phase transformations in the Mg-Ni-Ce system during MS and SPS, the following were established:

1. The successive use of MS and SPS allows for targeted changes in the phase composition and structure of the material.
2. An increase in the BPR, machining duration, and rotation speed accelerates the formation of the intermetallic compounds Mg_2_Ni, Mg_12_Ni_6_, and CeMg_3_.
3. At the final stage of SPS, the microstructure of the alloy is stabilized. Intermetallic phases are distributed uniformly, and the residual oxide content decreases.
4. For the Mg_2_Ni phase, the effective crystal size for storing hydrogen is 20–50 nm. In the case under study, after MS, the size of the Mg_2_Ni phase was 38.84 nm, and after SPS, it was 55.64 nm, which corresponds to the boundary of the optimal range.
5. The combination of MS and SPS helps to reduce the size of the crystal lattice. Preliminary MS ensures compositional homogeneity and activates diffusion processes, which are necessary for the formation of full-fledged intermetallic phases, whereas the absence of a mechanical treatment stage can lead to incomplete sintering and the formation of heterogeneous structures. Future studies may be aimed at a detailed assessment of the sorption characteristics of the alloys and their behavior in cyclic hydrogenation and dehydrogenation processes. Thus, the method of processing Mg-Ni-Ce alloys provides new prospects for the development of effective hydrogen absorption materials with high structural stability. Further studies may be aimed at a detailed assessment of the sorption characteristics of the obtained alloys and their behavior in cyclic hydrogenation and dehydrogenation processes.

## Figures and Tables

**Table 1:** Data on Mg-Ni-Ce-based PMs after MS.

**Batch**	**Alloy Composition**	**Number of Samples**	**BPR Groups**
I	Mg-3%Ni-2%Ce	12	10:1, 20:1, 30:1
II	Mg-7%Ni-3%Ce	12	10:1, 20:1, 30:1
III	Mg-10%Ni-5%Ce	12	10:1, 20:1, 30:1

**Table 2:** The average crystallite sizes of various phases after MS.

Phase	2θ (Degree)	FWHM (Degree)	Crystallite Size (nm)
Mg	36.2	0.28	29.85
Mg_2_Ni	43.2	0.22	38.84
Mg_12_Ni_6_	47.8	0.19	45.73
CeMg_12_	32.5	0.30	27.58
MgNi_2_	38.1	0.25	33.62

**Table 3:** Modification of the phase composition and structure of Mg-Ni-Ce by alloying with nickel and cerium.

Sample	Main Phases	Average Size of Crystals (nm)	Mg Content (at.%)	Ni Content (at.%)	Ce Content (at.%)
Mg-3%Ni-2%Ce	Mg·Mg_2_Ni·CeMg_12_	350	91.84	5.24	2.92
Mg-7%Ni-3%Ce	Mg·Mg_2_Ni·CeMg_3_	220	88.9	8.18	2.93
Mg-10%Ni-5%Ce	Mg·Mg_2_Ni·Ce_2_Mg_17_	60	69.95	23.18	6.87

## Data Availability

The original contributions presented in this study are included in the article. Further inquiries can be directed to the corresponding author.
